# The influence of hydration status on ion transport in the rabbit (*Oryctolagus cuniculus*) skin—An *in vitro* study

**DOI:** 10.1371/journal.pone.0255825

**Published:** 2021-08-12

**Authors:** Iga Hołyńska-Iwan, Paulina Smyk, Agnieszka Chrustek, Dorota Olszewska-Słonina, Karolina Szewczyk-Golec

**Affiliations:** 1 Department of Pathobiochemistry and Clinical Chemistry, Faculty of Pharmacy, Ludwik Rydygier Collegium Medicum in Bydgoszcz, Nicolaus Copernicus University, Toruń, Poland; 2 Department of Pediatric Nursing, Faculty of Health Sciences, Ludwik Rydygier Collegium Medicum in Bydgoszcz, Nicolaus Copernicus University, Toruń, Poland; 3 Department of Medical Biology and Biochemistry, Faculty of Medicine, Ludwik Rydygier Collegium Medicum in Bydgoszcz, Nicolaus Copernicus University, Toruń, Poland; National Institute of Child Health and Human Development (NICHD), NIH, UNITED STATES

## Abstract

The preservation of physiological transport of ions and water content is particularly important for maintaining the skin barrier, touch and pain stimuli, as well as the initiation of skin regeneration processes, especially after treatments associated with breaking skin continuity and wound healing difficulties. The aim of the study was to assess changes in ion transport, measured as values of transepithelial electric resistance and potential difference in stationary conditions and during mechanical-chemical stimulations, depending on the hydration status of isolated rabbit skin specimens. The specimens were divided into five groups: control (n = 22), dehydrated in 10% NaCl (n = 30), rehydrated after dehydration (n = 26), dried at 37°C (n = 26), and rehydrated after drying (n = 25). Dehydrated tissue samples showed altered resistance compared to the control; this change was maintained regardless of rehydration. In the dehydrated samples, changes in the measured electric potential were also noted, which returned to values comparable with the control after rehydration. Dehydrated skin, regardless of the cause of dehydration, responds with changes in the transport of sodium and chloride ions and the altered cellular microenvironment. It could influence the perception of stimuli, particularly pain, and slow down the regeneration processes.

## Introduction

The level of moisture and hydration of the skin is important for effective receptor stimulation, also by pain stimuli, as well as collagen and elastin production, skin regeneration and maintaining a healthy appearance of the skin [1–4]. A properly hydrated skin is a barrier for microorganisms and external factors, while retaining the ability of physiological exfoliation, and has the capacity of receiving external stimuli [5–10]. Dehydrated skin becomes scratchy, mat, flushed, prone to exfoliation, and the occurring micro-damage leads to increased permeability and influx and activation of immunocompetent cells [4, 9, 11–13]. Decreased water content causes accumulation of corneocytes on the epidermal surface and disrupts exfoliation [3, 5, 14]. A lower level of hydration of keratinocytes leads to a slower and, eventually, ceased production of collagen, as well as limited transfer of single fibers into the extracellular matrix [9, 15]. Similarly, the synthesis of other matrix proteins (i.e., elastin, keratin, laminin) and lipids (ceramides) first slows down and then ceases [3, 16]. The decreased amount of the flexible and water-binding fibers leads to changes in the permeability of the epidermal barrier, and consequently to disruption of the differentiation and regeneration processes [4, 9, 10, 14–16]. Changes in hydration are accompanied by impaired transport of ions, especially sodium [12, 13]. Maintaining physiological transport of water and ions is crucial for skin regeneration after treatments involving breaking skin continuity, as well as during the healing of complicated wounds, especially pressure ulcers, frostbites and burns [5, 9, 12, 13]. Moreover, proper hydration and the associated ion transport are important for the effectiveness of reconstructive, esthetic and cosmetic procedures. As a result of loss of water, skin layers lose their barrier properties against chemicals and microorganisms [5, 10, 14]. Altered composition of sweat causes its pH to be no longer acidic, as well as involves decreased quantities of NaCl and organic acids [4, 10]. Higher-pH sweat constitutes a weaker antimicrobial barrier, which is of critical importance in case of a break in the continuity of the skin, e.g., after surgical interventions [10]. Dehydrated skin is characterized by changes in the microbiome and increased colonization by certain microorganisms, e.g.,*Staphylococcus aureus* [9, 17].

Transport of ions in the skin is crucial for the correct functioning of the entire body, especially in conditions associated with an increased loss or uneven distribution of water [4, 18]. The increased transport of sodium ions through epithelial sodium channels (ENaCs) in keratinocytes can be associated with a release of low-molecular weight substances, which leads to the migration and activation of immunocompetent cells [2, 12, 13, 15]. Moreover, it has been shown that changes in ion transport affect antigen presentation and can lead to the activation of inflammation, which impairs tissue regeneration and wound healing [3, 11, 18].

Taking into account the relevance of the preservation of physiological transport of ions and water content for maintaining the skin barrier, touch and pain stimuli, as well as the initiation of skin regeneration processes, the studies on the transepithelial ion transport pathways in different hydration conditions seem to be of high importance [19–22]. However, the avalaible data concerning these processes in the full-thickness skin with preserved layered structure and nerve endings are still sparse. It could be assumed that the ion transport would be changed as a result of different dehydration methods. Therefore, the aim of the study was to assess the transepithelial transport of sodium and chloride ions depending on the degree of hydration of isolated skin fragments. The fragments were dehydrated, and changes in electric resistance and transmembrane electric potential were assessed in stationary conditions, as well as during mechanical stimulation with iso-osmotic Ringer’s solution (RH) and mechanical–chemical stimulation with solutions containing sodium and chloride transmembrane transport inhibitors. The electrophysiological properties of the skin samples after rehydration were also investigated.

## Materials and methods

The measurements of electrophysiological parameters were conducted on skin specimens derived from the abdomens of 10 rabbits in a modified Ussing chamber [23, 24]. The experiments were approved by Local Committee for Ethical Animal Experiments of the Universities of Bydgoszcz (permit No. 16/21012, June 21, 2012).

### Study design

The experiment was conducted using 129 isolated skin fragments taken from 10 albino New Zealand rabbits aged 2–3 months of both sexes, weighing 3.5–4.0 kg. The animals were purchased from the Department of Experimental Medicine, Silesia Medical University, Katowice, Poland. The animals were maintained on a 12/12 light/dark cycle and 50% average humidity, with food (certified laboratory forage for rabbits, Labofeed KB, Morawski inc. Kcynia, Poland) and water available *ad libitum*. Two rabbits were housed in one cage (size: 1.19 x 0.58 x 0.61 m, with a shelf). The animals were asphyxiated with carbon dioxide (60% in inhaled air). In every case, the animal death was confirmed through a direct cardiac palpation. The material was sampled from abdominal skin, with hair removed mechanically. After the skin part was cut out, it was divided into fragments of approximately 2 cm^2^ and placed immediately in the RH. Then the skin fragments were divided into five groups and treated as follows:

1. Ctr—control (n = 22): specimens incubated in RH at room temperature for 30 min
2. Deh—dehydrated (n = 30): specimens incubated in 10% NaCl in RH for 30 min
3. RDeh—rehydrated after dehydration (n = 26): specimens incubated in RH for 30 min after dehydration in 10% NaCl for 30 min
4. Dr—dried (n = 26): specimens dried at 37°C for 60 min
5. RDr—rehydrated after drying (n = 25): specimens incubated in RH for 60 min after drying at 37°C for 60 min.

**Fig 1:**
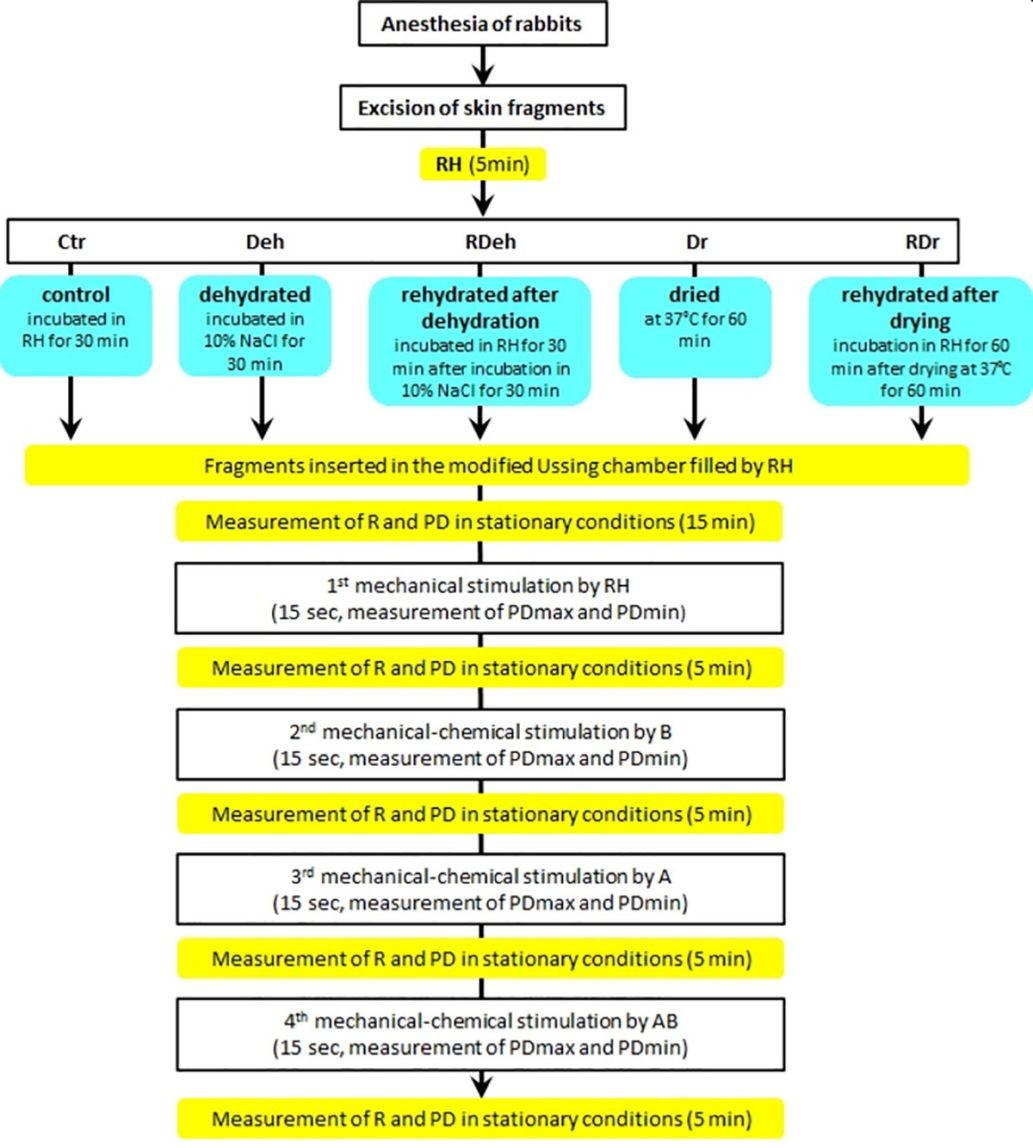
Study design. Abbreviations: RH–Ringer’s solution; NaCl–sodium chloride 10% (1.71 M) in RH; B–bumetanide solution (0.1 mM), used as a blocker of the Na^+^–K^+^–Cl^–^–cotransporter; A–amiloride solution (0.1 mM), used as blocker of ENaC (epithelial sodium channel); AB–solution of amiloride (0.1 mM) and bumetanide (0.1 mM); PD–transepithelial potential difference of the skin specimens measured in stationary conditions (mV); PDmax–maximal transepithelial potential measured during a 15–sec stimulation of the skin specimens (mV); PDmin–minimal transepithelial potential measured during a 15–sec stimulation of the skin specimens (mV); R–resistance (Ω/cm^2^).

Subsequently, the studied area of the tissue sample was 1 cm^2^. The electrophysiological parameters of pre-treated skin samples were measured in stationary conditions and during stimulations. The specimens were mounted horizontally in a modified Ussing chamber. The modification allowed stimulation of the skin surface with fluid using a peristaltic pump with a flow of 0.05 ml/s (0.75 ml/15 s). The lowering of free drops from a distance of 5 mm on skin surface was assumed as a mechanical stimulation. After the transepithelial potential (PD) was stabilized for the fragment, series of mechanical (with RH) and mechanical–chemical stimulation (with the solutions of ion transport inhibitors) were applied (Fig 1). The measured parameters were obtained as described previously [23, 24].

#### Chemicals and solutions

The following chemicals and solutions were used in the experiment. The preparation of RH, A (0.1 mM amiloride), B (0.1 mM bumetanide), and AB (0.1 mM amiloride and 0.1 mM bumetanide) solutions was described previously [25]. Sodium chloride (1.71 M) solution in RH was use as a hypertonic 10% NaCl solution.

Mineral compounds, namely KCl, NaCl, CaCl_2_, MgCl_2_, were purchased from POCH (Poland), and amiloride, bumetanide, Hepes and DMSO were purchased in Sigma-Aldrich (USA).

### Data analysis

The apparatus to measurement the voltage and resistance was connected to the data acquisition system MP150 which transferred the obtained data to the computer data acquisition software AcqKnowledge 3.8.1 (Biopac Systems, Inc., USA). Data were recorded following the experimental protocol EVC 4000 (WPI, USA).

The results were summarized in tables and figures. Statistical analysis was conducted in the Statistica 11.00 software (StatSoft, Inc.).

To calculate the sample size, the power of 80% and alpha level of 0.05 were used. The normality of data was checked using the Kolmogorov-Smirnov test.The Wilcoxon test was used to compare data from the same incubation conditions with the statistical significance level at p< 0.05. The Mann-Whitney test was used to detect significant differences (p<0.05) for the different experimental conditions in the various groups of tissues.

## Results

### Electrophysiological parameters measured during stationary conditions

#### Transmembrane electric resistance

**Fig 2:**
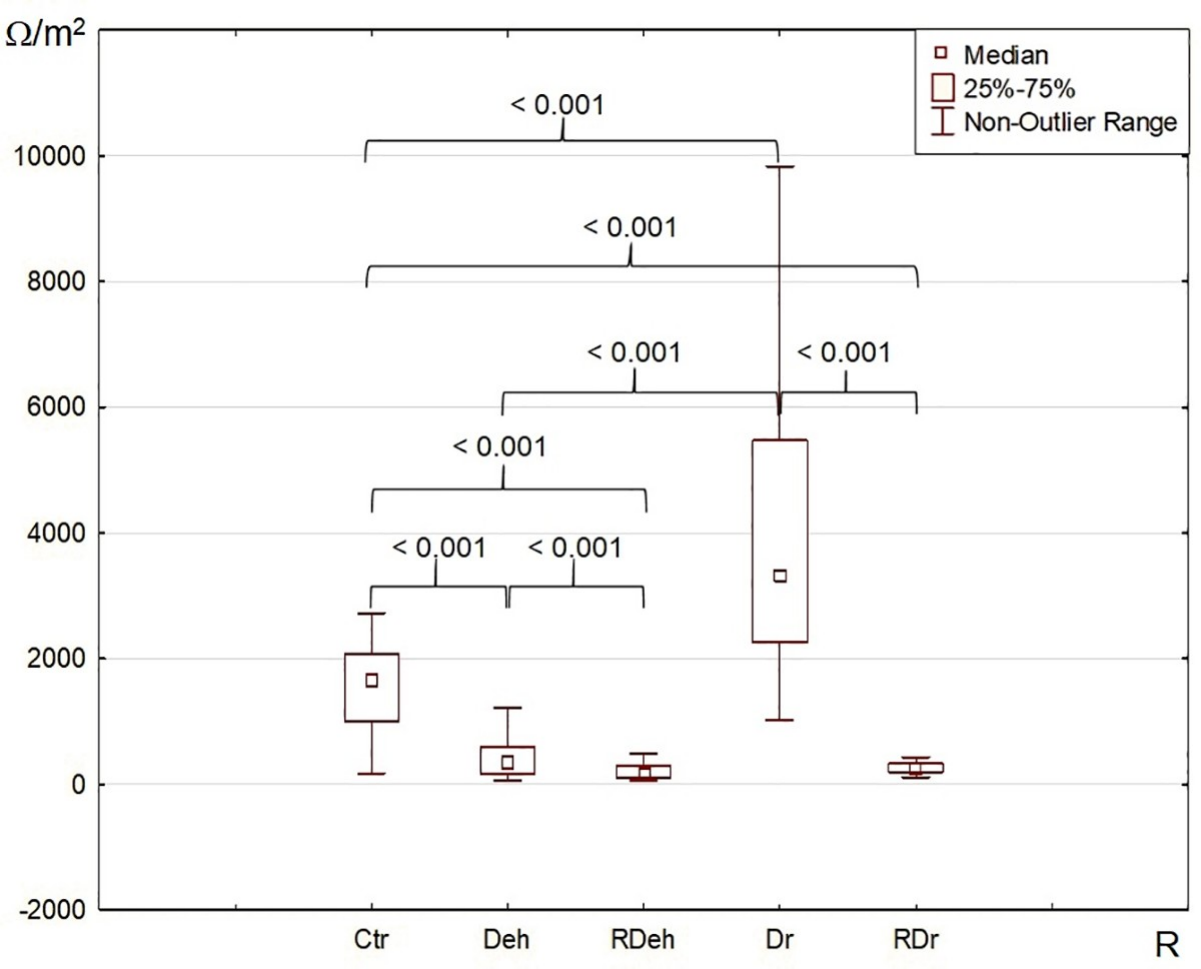
The transepithelial resistance (R, Ω/cm^2^) measured in stationary conditions of the isolated rabbit skin specimens incubated in Ringer’s solution (RH), pre–treated as follows: Ctr (control: skin specimens incubated in RH for 30 min), Deh (dehydrated: skin specimens incubated in 10% NaCl for 30 min), RDeh (rehydrated after dehydration: skin specimens rehydrated in RH for 30 min after incubation in 10% NaCl for 30 min), Dr (dried: skin specimens dried at 37°C for 60 min), RDr (rehydrated after drying: skin specimens rehydrated in RH for 60 min after drying at 37°C for 60 min). The p values < 0.05 were considered as statistically significant.

The median transmembrane electric resistance of the skin fragments incubated in Ringer’s solution (RH) (control group, Ctr) was 1672 Ω/cm^2^. The dried specimens (Dr) had the highest recorded median resistance of 3314 Ω/cm^2^. Tissue samples dehydrated with a 10% NaCl solution (Deh) had a median resistance of 352 Ω/cm^2^, and this value was lower in a statistically significant manner compared to Ctr and Dr. The skin samples rehydrated after dehydration (RDeh) or rehydrated after drying (RDr) had lower resistance values in comparison with Ctr, Deh and Dr (Fig 2, S1 Table). Changes in transmembrane electric resistance in the investigated tissue groups indicate alterations in the integrity of the skin and in the adherence of skin cells to each other. A stronger stimulus affecting the integrity and adherence of cells seems to be dehydration caused by a hypertonic solution.

#### Transepithelial electric potential

**Fig 3:**
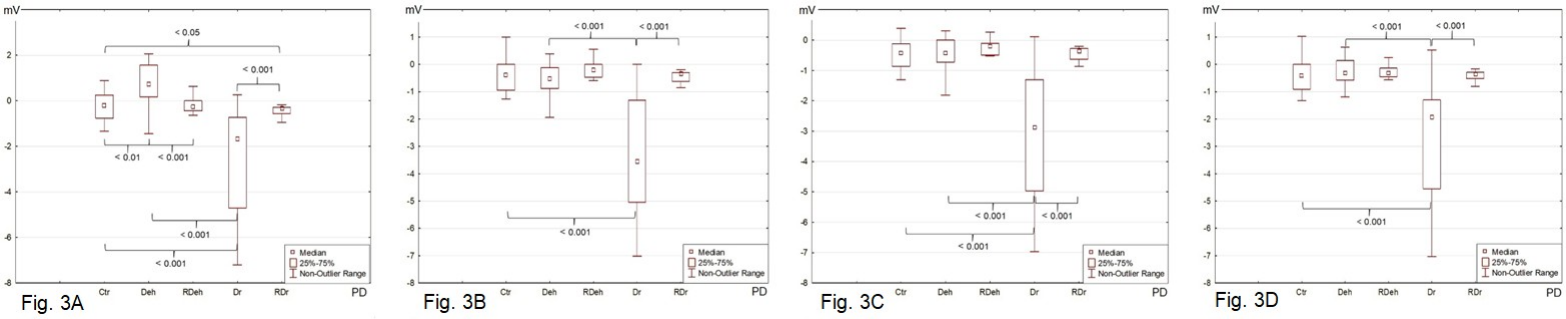
**The transepithelial electric potential (PD, mV) measured in stationary conditions of the isolated rabbit skin specimens incubated in**: (**a**) Ringer’s solution (RH); (**b**) bumetanide (0.1 mM) solution; (**c**) amiloride (0.1 mM) solution; (**d**) solution of amiloride (0.1 mM) and bumetanide (0.1 mM). The skin specimens were pre–treated as follows: Ctr (control: skin specimens incubated in RH for 30 min), Deh (dehydrated: skin specimens incubated in 10% NaCl for 30 min), RDeh (rehydrated after dehydration: skin specimens rehydrated in RH for 30 min after incubation in 10% NaCl for 30 min), Dr (dried: skin specimens dried at 37°C for 60 min), RDr (rehydrated after drying: skin specimens rehydrated in RH for 60 min after drying at 37°C for 60 min). The p values < 0.05 were considered as statistically significant.

Measurement of transepithelial electric potential in stationary conditions (PD) revealed the presence of transepithelial ion transport. The median of PD for Ctr was -0.19 mV, for Deh 0.74 mV, while for Dr -1.68 mV (Fig 3A, S1 Table). After the treatment with the use of chloride and sodium ion inhibitors, the skin fragments responded repeatedly with a decreased PD that would not come back to the pre-stimulation values (Fig 3B–3D). The RDr fragments showed a potential similar to that of Ctr. The obtained parameters differed in a statistically significant manner, but drying affected the transport of sodium and chloride ions more potently than dehydration (Fig 3, S3–S6 Tables).

### Electrophysiological parameters measured during stimulation conditions

#### Mechanical stimulation

**Fig 4:**
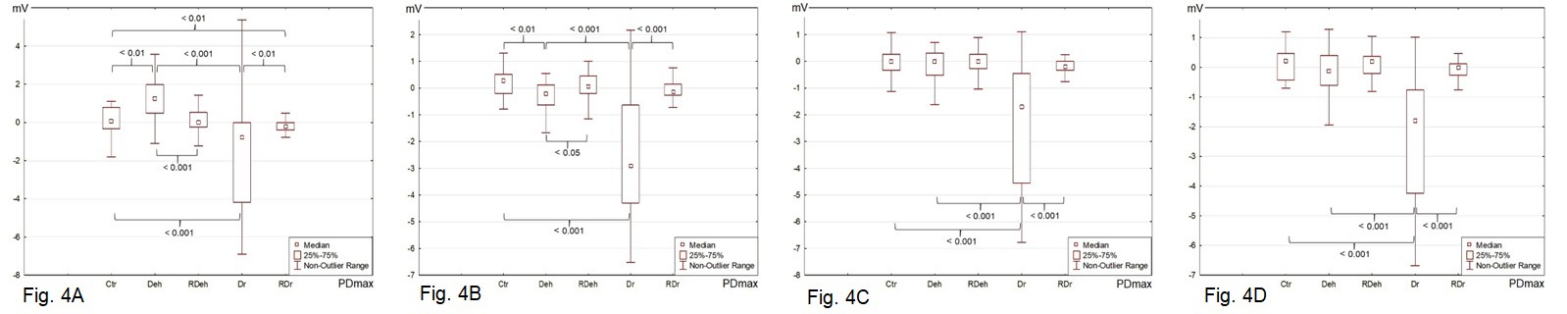
**The maximal transepithelial electric potential (PDmax, mV) measured in 15s mechanical–chemical stimulation of the isolated rabbit skin specimens with the use of**: **(a)** Ringer’s solution (RH); **(b)** bumetanide (0.1 mM) solution; **(c)** amiloride (0.1 mM) solution; **(d)** solution of amiloride (0.1 mM) and bumetanide (0.1 mM). The skin specimens were pre–treated as follows: Ctr (control: skin specimens incubated in RH for 30 min), Deh (dehydrated: skin specimens incubated in 10% NaCl for 30 min), RDeh (rehydrated after dehydration: skin specimens rehydrated in RH for 30 min after incubation in 10% NaCl for 30 min), Dr (dried: skin specimens dried at 37°C for 60 min), RDr (rehydrated after drying: skin specimens rehydrated in RH for 60 min after drying at 37°C for 60 min). The p values < 0.05 were considered as statistically significant.

**Fig 5:**
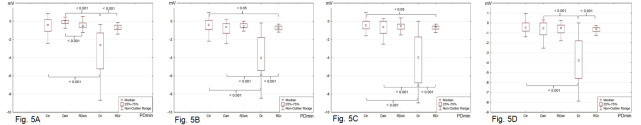
**The minimal transepithelial electric potential (PDmin, mV) measured in 15s mechanical–chemical stimulation of the isolated rabbit skin specimens with the use of**: (**a**) Ringer’s solution (RH); (**b**) bumetanide (0.1 mM) solution; (**c**) amiloride (0.1 mM) solution; (**d**) solution of amiloride (0.1 mM) and bumetanide (0.1 mM). The skin specimens were pre–treated as follows: Ctr (control: skin specimens incubated in RH for 30 min), Deh (dehydrated: skin specimens incubated in 10% NaCl for 30 min), RDeh (rehydrated after dehydration: skin specimens rehydrated in RH for 30 min after incubation in 10% NaCl for 30 min), Dr (dried: skin specimens dried at 37°C for 60 min), RDr (rehydrated after drying: skin specimens rehydrated in RH for 60 min after drying at 37°C for 60 min). The p values < 0.05 were considered as statistically significant.

Mechanical stimulation with RH resulted in repeated changes in ion transport measured as PDmax and PDmin during the 15-sec pacing. Stimulation with RH induced a median PDmax of -0.06 mV and a median PDmin of -0.33 mV for the Ctr group. The Deh group had a median PDmax of 1.25 mV and a median PDmin of -0.78 mV, whereas the Dr group had a median PDmax of -0.78 mV and a median PDmin of -2.59 mV (Figs 4A and 5A, S2 Table). All measured PDmax and PDmin values were significantly different from the PD measured in stationary conditions for each investigated group (Wilcoxon test, S3–S6 Tables). This suggests the occurrence of a repetitive change in ion transport during mechanical stimulation. Ctr showed the smallest changes in potential and were the least responsive. Dr showed a significantly increased ion flow, similarly to Deh.

#### Mechanical-chemical stimulation

Mechanical–chemical stimulation resulted in repeated changes in ion transport measured as PDmax and PDmin during the 15-sec pacing. The use of blockers of transepithelial routes of chloride (bumetanide, B) (Fig 4B) and sodium (amiloride, A) (Fig 4C) ion transport, also in combination (the solution of amiloride and bumetanide, AB) (Fig 4D), showed a response different in a statistically significant manner for the Ctr, Deh and Dr fragments, analogous to that observed after mechanical stimulation with RH.

Rehydration of the skin samples after both dehydration and drying caused significant changes in PDmax and PDmin (Figs 4 and 5). The PDmax and PDmin values of RDeh and RDr were comparable to the values observed in Ctr. The response was similar regardless of the experimental group. The inhibitors of flow of chloride, sodium, and concurrently sodium and chloride ions caused a decrease in the transport of the appropriate ions, which was reflected by the decrease in the measured PDmax and PDmin values.

## Discussion

Dry skin is not only an esthetic, but also a medical problem, which is extremely important in cases involving breaking skin continuity in dehydrated patients or in cases of skin exposure to hypertonic medicines/substances [5, 11, 14]. In the study, we proposed two methods of dehydration of skin samples prepared in a way that conserved the layered tissue structure. These tissue fragments contained keratinocytes (95%), corneocytes, fibroblasts, immunocompetent cells, hair follicles and nerve fiber terminals [19, 21]. Because of its layered structure, the mammalian skin is naturally protected from drying [3, 5]. Therefore, the observed increase in resistance suggests an increased adherence and retained continuity of the investigated tissue surface [16, 19, 20].

Changes in the hydration of the skin are associated with a loss of water from the cells and the extracellular space and can include individual and/or all skin layers [3–5, 14, 20, 21]. Skin dehydration involves hyperreactivity to pain due to more intensive transport of sodium ions from keratinocytes into the extracellular space and stimulation of nerve terminals, as well as exacerbation of symptoms in atopic dermatitis [4, 6, 22]. In the layers of dehydrated skin, excitation lasts longer due to the inability to dilute the accumulated sodium ions. Their uptake by the cell is limited and associated with the onset of a hypertonic gradient and water loss. The presented results (Figs 3–5, S1 and S2 Tables) show an increased transport of sodium ions in the Deh and Dr skin samples. Concurrently, skin specimens with a physiological water content held in the iso-osmotic RH were not reactive, and the ion transport processes occurred less intensively (Figs 4 and 5). The most prominent changes concerned the transport of sodium ions (response to solution A), while increased secretion of chloride ions (in response to solution B) was observed only for the Deh skin samples. A properly moisturized skin parts did not show any significant response to mechanical and mechanical–chemical stimulation. A similar intensification of the transport of sodium ions has been observed in other studies in which the irritant substance, capsaicin, was applied on the prepared skin sample, leading to the release of neuromediators from nerve endings and thus acting analgesically [23]. However, the obtained PD values were significantly lower than those recorded by Barker at al [26]. In their research, the potential was 6 +/- 7 mV, measured on the abdomen of guinea pigs. However, they used a different research model, measuring the skin potential of a living, properly hydrated animal [26]. The model presented in our research was based on prepared skin fragments with removed subcutaneous tissue. Thus the skin samples were deprived of access to nutrients and oxygen. A limited ATP pool was available, which significantly slowed down the metabolic processes and ion transport. However, the potential values of Deh and Dr skin samples may indicate more intense ion transport processes than in Ctr, RDeh and RDr tissue samples.

It should be noted that the PD values for the skin fragments subjected to drying and dehydration were similar to the values described by Denda et al. [7]. The authors used a model of hairless mice and proved that the measured transepithelial potential was primarily influenced by the tissue microenvironment and neuroendocrine factors that directly modify the transport of sodium and potassium ions [7]. The applied drying and dehydration processes significantly influenced the hydration of the tested cells and caused changes in the microenvironment of the analyzed fragments. For the control fragments, the PD values remained at the level of -0.19 to -0.43 mV and were significantly higher than the tissue samples subjected to the drying process and those obtained by Denda et al. [7]. It could be concluded that the measured potential of control tissue specimens immersed in an isotonic solution resulted directly from the ability to transport sodium and chloride ions. No changes in the hydration of intercellular spaces and the cells themselves interfered with this process.

The transport of sodium ions in keratinocytes is associated with the regeneration processes [13, 15]. Changes in the flow of sodium ions during dehydration can be especially dangerous for patients with a broken skin continuity, wounds following surgical treatment, burns and healing thereof, as well as pressure ulcers [9, 12]. It has been proven that changes in the transport of sodium ions occurring in keratinocytes can be associated with the release of low-molecular weight substances, especially proinflammatory agents [2, 6, 9, 11]. It can have an impact on the activation and migration of immunocompetent cells and the initiation of inflammatory reactions [2, 11]. The migration of immunocompetent cells and the development of inflammatory response is associated with the onset of hypersensitivity and/or allergies [6, 12–13, 15]. In the presented study, it was shown that skin dehydration using a NaCl solution is a stronger stimulus for an increased transport of sodium ions than drying at 37°C. Skin fragments incubated in a hypertonic environment were characterized by a decrease in electric potential (Figs 4 and 5, S2 Table) due to changes in the absorption of sodium ions and the secretion of chloride ions. Such a decrease was not observed for fragments incubated at 37°C, which confirmed that the layered skin structure provided protection against water evaporation. The transport of sodium and chloride ions in the skin subjected to drying was altered to a small extent. From a practical point of view, in the case of surgical procedures, it is important to maintain the appropriate level of hydration in the patient and not to conduct the procedures if skin dehydration is observed. Moreover, due to the possibility of problems with regeneration, pain hyperreactivity and development of inflammation, avoiding the exposure to dehydration is important for the regeneration processes in the skin after treatments associated with breaking its continuity and during treatment of inflammatory conditions [3, 6, 11, 21, 22]. Incubation of the Deh and Dr skin samples in iso-osmotic RH resulted in a partial reversal of the potential changes. However, in the RDr specimens, ion transport did not return to the values comparable to Ctr after the use of bumetanide (Figs 4 and 5). The drying stimulus seems to interfere with ion transport stronger than dehydration in hypertonic solution.

High electric resistance in the skin reflects its physiological condition, health status and the content of each layer, as well as the degree of preservation of intercellular junctions and ability to bind water by the appropriate proteins released into the extracellular matrix [1, 13, 20, 23–25]. The electric resistance of the skin depends on the integrity of the epidermis, preservation of tight junctions and activity of sweat glands, which is associated with maintaining the appropriate hydration level [1, 20]. In the presented study, skin fragments with the proper degree of hydration (Ctr) exhibited lower resistance values compared with skin fragments subjected to drying (Dr), while the highest resistance values were recorded for Deh (Fig 2, S1 Table). Additionally, the resistance values of the Deh and Dr samples that were rehydrated in the RH solution (RDeh and RDr) remained statistically decreased when compared to Ctr (Fig 2). As a result of persistent dehydration, the structure of the skin becomes damaged, which is accompanied by a decrease in electric resistance [20]. Changes in skin hydration cause water molecules to detach from keratin, collagen and elastin fibrils, which in turn causes their rupture and accelerated degradation [5, 9, 14, 27]. Layer of the skin with a reduced number of fibrils is unable to deform, which is the cause of microdamage [5, 25, 27]. The activity of proteolytic enzymes as a result of the reduced water content is limited to small regions located around cells, hence enzymatic degradation is more intensive during serious dehydration, in the presence of the reduced amount of free water molecules [5, 9, 14, 21]. The problem of deformation and microdamage is associated with a high electric resistance of the skin, although its values after cell separation and the occurrence of microruptures decrease [20]. Dehydrated cells, non-elastic collagen and elastin fibers with tendency to rupture, as well as uncontrolled degradation of barrier proteins and thick extracellular matrix with spaces of varying enzymatic activities, initiate local reactions leading to the formation of sulci and wrinkles [3, 9, 14, 25, 27]. What is more, the barrier function of the skin is influenced by endocrine factors, in particular cortisol [8]. It has been proven that skin permeability and the mechanism of restoring tightness decrease after the use of stress stimuli, which is related to changes in the production of proteins by keratinocytes under the influence of cortisol [8].

The major limitation of the study is the difficulty of translating the results obtained easily from animals to humans. Some properties of rabbit skin, including a thickness, as well as nervous, hormonal and immunological regulation may differ from human tissue, but the characteristic of rabbit skin makes it more similar to human skin than in the case of other experimental animals, including guinea pigs or mice [5, 21, 26]. However, an access to human tissues from healthy individuals is extremely difficult and there are no alternative tissue cultures that would show the same features as in human skin. No grafts have been obtained with an adequate number and arrangement of cells, multilayered structure, the proper composition of intercellular spaces and nerve endings. Studies on the skin tissue collected from healthy rabbits makes it possible to assess changes in ion transport in selected experimental conditions in the tissue with preserved layering, reactivity and permeability for xenobiotics.

## Conclusions

The applied dehydration and drying conditions caused rehydration-persistent changes in the transepithelial electrical resistance, related to irreversible changes in the cellular microenvironment as a result of dehydration. Deficiency of water in the environment surrounding the cells resulted in more intensive movement of sodium ions into cells and chloride ions from cells. These changes were reversible due to rehydration of the samples.

The demonstrated changes in the electrophysiological properties of the skin should be taken under consideration in patients at risk of skin dehydration. The observed intensification in the transport of sodium ions through dehydrated skin cells could influence the perception of stimuli, particularly pain, and slow down the regeneration processes or even give rise to overreactivity and allergy. Therefore, in patients with a broken skin continuity and those undergoing skin care procedures, the skin dehydration should be avoided and properly treated.

## Supporting information

S1 Table The values of transepithelial Potential Difference (PD) and electric Resistance (R) measured in stationary conditions for the analyzed skin samples. (DOCX)

S2 Table The values of transepithelial potential difference (PDmax, PDmin) measured during 15s stimulations for the analyzed skin samples. (DOCX)

S3 Table The Wilcoxon test p values for mechanical stimulation by Ringer’s solution of analyzed skin samples. (DOCX)

S4 Table The Wilcoxon test p values for mechanical-chemical stimulation by bumetanide (0.1 mM) solution of analyzed skin samples. (DOCX)

S5 Table The Wilcoxon test p values for mechanical-chemical stimulation by amiloride (0.1 mM) solution of analyzed skin samples. (DOCX)

S6 Table The Wilcoxon test p values for mechanical-chemical stimulation by the solution of amiloride (0.1 mM) and bumetanide (0.1 mM) of analyzed skin samples. (DOCX)

S1 Graphical abstract (DOCX)

## Abbreviations

A: amiloride solution (0.1 mM)
B: bumetanide solution (0.1 mM)
AB: solution of amiloride (0.1 mM) and bumetanide (0.1 mM)
CFTR: cystic fibrosis transmembrane regulator
ENaC: epithelial sodium channel
NKCC: Na^+^-K^+^-Cl^-^ cotransporter
PD: transepithelial potential difference measured in stationary conditions
PDmin: minimal transepithelial potential difference measured during 15s stimulation
PDmax: maximal transepithelial potential difference measured during 15s stimulation
R: transepithelial electrical resistance measured in stationary conditions
RH: iso-osmotic Ringer’s solution
Ctr: Control: skin specimens incubated in RH for 30 min
Deh: Dehydrated: skin specimens incubated in 10% NaCl for 30 min
RDeh: Rehydrated after dehydration: skin specimens rehydrated in RH for 30 min after dehydration in 10% NaCl for 30 min
Dr: Dried: skin specimens dried at 37°C for 60 min
RDr: Rehydrated after drying: skin specimens rehydrated in RH for 60 min after drying at 37°C for 60 min
