# Supplementary material for: The influence of hydration status on ion transport in the rabbit (Oryctolagus cuniculus) skin—An in vitro study
Source: PLoS One. 2021 Aug 12;16(8):e0255825. doi: 10.1371/journal.pone.0255825 (PMC8360594; doi:10.1371/journal.pone.0255825)
Supplement: S1 Table — (DOCX) [file pone.0255825.s001.docx]

**S1 Table.** The values of transepithelial potential difference (PD) and electric resistance (R) measured in stationary conditions for the analyzed skin samples.

| Conditions | | **Ctr**  (n=22) | | | | **Deh**  (n=30) | | | | **RDeh**  (n=26) | | | | **Dr**  (n-26) | | | | **RDr**  (n=25) | | | |  |
| --- | --- | --- | --- | --- | --- | --- | --- | --- | --- | --- | --- | --- | --- | --- | --- | --- | --- | --- | --- | --- | --- | --- |
| Parameters | | **RH** | **B** | **A** | **AB** | **RH** | **B** | **A** | **AB** | **RH** | **B** | **A** | **AB** | **RH** | **B** | **A** | **AB** | **RH** | **B** | **A** | **AB** | |
| PD | median | -0.19 | -0.39 | -0.43 | -0.41 | 0.74 | -0.51 | -0.43 | -0.32 | -0.25 | -0.2 | -0.21 | -0.31 | -1.68 | -3.56 | -2.88 | -1.94 | -0.34 | -0.35 | -0.36 | -0.35 | |
|  | upper quartile | -0.76 | -0.95 | -0.86 | -0.91 | 0.17 | -0.88 | -0.73 | -0.58 | -0.57 | -0.62 | -0.63 | -0.52 | -4.72 | -5.04 | -4.97 | -4.55 | -0.44 | -0.47 | -0.5 | -0.45 | |
|  | lower quartile | 0.25 | 0.00 | -0.12 | 0.00 | 1.56 | -0.12 | 0.00 | 0.14 | -0.29 | -0.3 | -0.28 | -0.29 | -0.72 | -1.31 | -1.30 | -1.30 | 0 | 0 | -0.11 | -0.14 | |
| R | median | 1672 | | | | 352 | | | | 151 | | | | 3314 | | | | 244 | | | | |
|  | upper quartile | 992 | | | | 155 | | | | 199 | | | | 2255 | | | | 93 | | | | |
|  | lower quartile | 2063 | | | | 593 | | | | 327 | | | | 547 | | | | 302 | | | | |

Abbreviations: Ctr - control: skin specimens incubated in RH for 30 min; Deh - dehydrated: skin specimens incubated in 10% NaCl for 30 min; RDeh - rehydrated after dehydration: skin specimens rehydrated in RH for 30 min after incubation in 10% NaCl for 30 min; Dr - dried: skin specimens dried at 37°C for 60 min; RDr - rehydrated after drying: skin specimens rehydrated in RH for 60 min after drying at 37°C for 60 min; RH - Ringer’s solution; B - bumetanide (0.1 mM) solution; A - amiloride (0.1 mM) solution; AB - solution of amiloride (0.1 mM) and bumetanide (0.1 mM); PD - transepithelial potential difference of the skin specimens measured in stationary conditions (mV); R - resistance (Ω/cm^2^).
