# Supplementary material for: The influence of hydration status on ion transport in the rabbit (Oryctolagus cuniculus) skin—An in vitro study
Source: PLoS One. 2021 Aug 12;16(8):e0255825. doi: 10.1371/journal.pone.0255825 (PMC8360594; doi:10.1371/journal.pone.0255825)
Supplement: S2 Table — (DOCX) [file pone.0255825.s002.docx]

**S2 Table.** The values of transepithelial potential difference (PDmax, PDmin) measured during 15s stimulations for the analyzed skin samples.

| Conditions | | **Ctr**  (n=22) | | | | **Deh**  (n=30) | | | | **RDeh**  (n=26) | | | | **Dr**  (n=26) | | | | **RDr**  (n=25) | | | |
| --- | --- | --- | --- | --- | --- | --- | --- | --- | --- | --- | --- | --- | --- | --- | --- | --- | --- | --- | --- | --- | --- |
| Parameters | | **RH** | **B** | **A** | **AB** | **RH** | **B** | **A** | **AB** | **RH** | **B** | **A** | **AB** | **RH** | **B** | **A** | **AB** | **RH** | **B** | **A** | **AB** |
| PDmax | median | 0.06 | 0.27 | 0.00 | 0.21 | 1.25 | -0.11 | 0.00 | -0.12 | 0 | 0.06 | 0 | 0.19 | -0.78 | -2.92 | -1.71 | -1.80 | -0.21 | -0.13 | -0.21 | 0 |
|  | upper quartile | -0.34 | -0.21 | -0.34 | -0.43 | 0.49 | -0.64 | -0.52 | -0.61 | -0.4 | -0.27 | -0.34 | -0.27 | -4.18 | -4.30 | -4.55 | -4.24 | -0.24 | -0.21 | -0.27 | -0.21 |
|  | lower quartile | 0.79 | 0.52 | 0.27 | 0.46 | 1.98 | 0.12 | 0.31 | 0.40 | 0 | 0.15 | 0 | 0.12 | 0.00 | -0.64 | -0.46 | -0.76 | 0.52 | 0.46 | 0.27 | 0.37 |
| PDmin | median | -0.33 | -0.39 | -0.45 | -0.45 | 0.00 | -0.63 | -0.61 | -0.55 | -0.49 | -0.58 | -0.53 | -0.5 | -2.59 | -4.02 | -3.97 | -3.77 | -0.68 | -0.73 | -0.64 | -0.62 |
|  | upper quartile | -1.04 | -0.89 | -0.82 | -0.82 | -0.24 | -1.31 | -1.31 | -1.19 | -0.89 | -0.92 | -0.85 | -0.85 | -5.22 | -5.4 | -6.73 | -5.58 | -0.67 | -0.7 | -0.85 | -0.98 |
|  | lower quartile | 0.18 | 0.12 | 0.00 | 0.00 | 0.12 | -0.27 | 0.00 | 0.00 | -0.43 | -0.52 | -0.52 | -0.46 | -1.28 | -1.77 | -1.71 | -1.80 | -0.18 | -0.22 | -0.31 | -0.21 |

Abbreviations: Ctr - control: skin specimens incubated in RH for 30 min; Deh - dehydrated: skin specimens incubated in 10% NaCl for 30 min; RDeh - rehydrated after dehydration: skin specimens rehydrated in RH for 30 min after incubation in 10% NaCl for 30 min; Dr - dried: skin specimens dried at 37°C for 60 min; RDr - rehydrated after drying: skin specimens rehydrated in RH for 60 min after drying at 37°C for 60 min; RH - Ringer’s solution; B - bumetanide (0.1 mM) solution; A - amiloride (0.1 mM) solution; AB - solution of amiloride (0.1 mM) and bumetanide (0.1 mM); PDmax - maximal transepithelial potential measured during a 15-sec stimulation of the skin specimens (mV); PDmin - minimal transepithelial potential measured during a 15-sec stimulation of the skin specimens (mV).
