# Supplementary material for: The influence of hydration status on ion transport in the rabbit (Oryctolagus cuniculus) skin—An in vitro study
Source: PLoS One. 2021 Aug 12;16(8):e0255825. doi: 10.1371/journal.pone.0255825 (PMC8360594; doi:10.1371/journal.pone.0255825)
Supplement: S3 Table — (DOCX) [file pone.0255825.s003.docx]

**S3 Table.** The Wilcoxon test p values for mechanical stimulation by Ringer’s solution of analyzed skin samples.

|  | **Ctr**  (n=22) | **Deh**  (n=30) | **RDeh**  (n=26) | **Dr**  (n=26) | **RDr**  (n=25) |
| --- | --- | --- | --- | --- | --- |
| PD vs PDmin | *<0.001* | *<0.001* | *<0.001* | *<0.001* | *<0.001* |
| PD vs PDmax | *<0.001* | *<0.001* | *<0.001* | *<0.05* | *<0.001* |
| PDmin vs PDmax | *<0.01* | *<0.001* | *<0.001* | *0.016* | *<0.001* |

Abbreviations: Ctr - control: skin specimens incubated in Ringer’s solution for 30 min; Deh - dehydrated: skin specimens incubated in 10% NaCl for 30 min; RDeh - rehydrated after dehydration: skin specimens rehydrated in RH for 30 min after incubation in 10% NaCl for 30 min; Dr - dried: skin specimens dried at 37°C for 60 min; RDr - rehydrated after drying: skin specimens rehydrated in RH for 60 min after drying at 37°C for 60 min; PD - transepithelial potential difference of the skin specimens measured in stationary conditions (mV); PDmax - maximal transepithelial potential measured during a 15-sec stimulation of the skin specimens (mV); PDmin - minimal transepithelial potential measured during a 15-sec stimulation of the skin specimens (mV).
