# Supplementary figures and images for: The influence of hydration status on ion transport in the rabbit (Oryctolagus cuniculus) skin—An in vitro study
Source: PLoS One. 2021 Aug 12;16(8):e0255825. doi: 10.1371/journal.pone.0255825 (PMC8360594; doi:10.1371/journal.pone.0255825)

**S1 Graphical abstract**

**
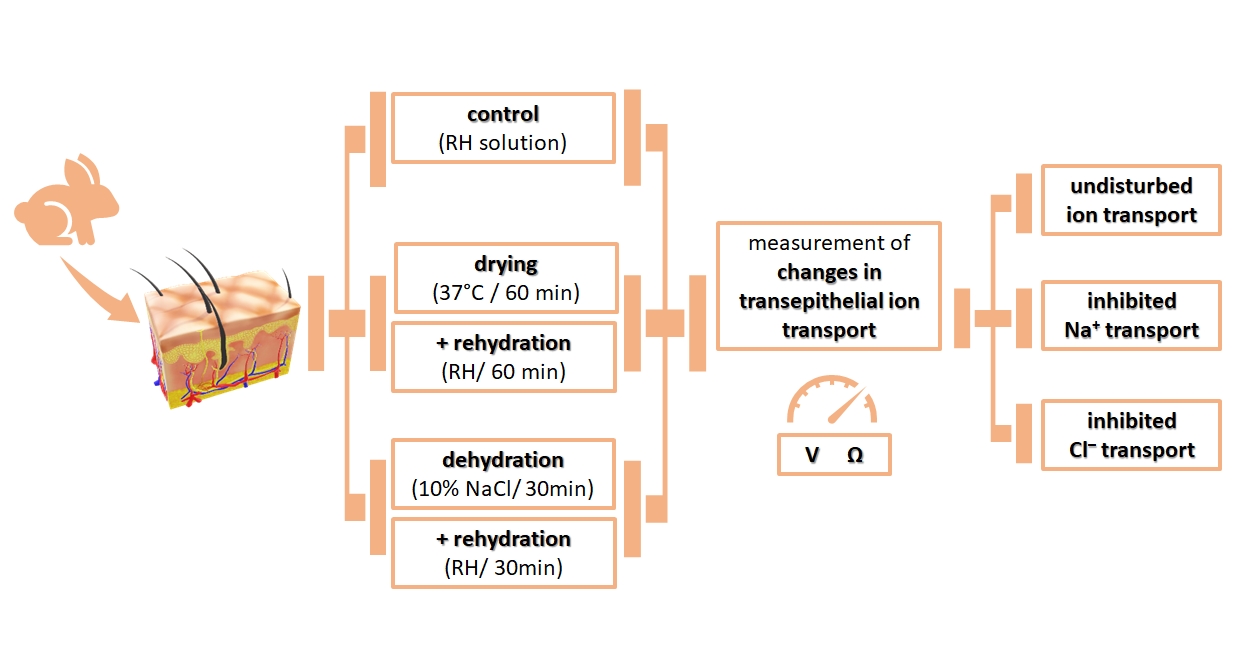
**

Supplement: S1 Graphical abstract — (DOCX) [file pone.0255825.s007.docx]
